# Operationalizing the Global Leadership Initiative in Sarcopenia: Muscle‐Specific Strength, Optimal Criteria and Clinical Relevance

**DOI:** 10.1002/jcsm.70222

**Published:** 2026-01-30

**Authors:** Liangyu Yin, Yu Cao, Mengda Tang, Hanping Shi, Hua Jiang, Jinghong Zhao

**Affiliations:** ^1^ Department of Nephrology, Chongqing Key Laboratory of Prevention and Treatment of Kidney Disease, Chongqing Clinical Research Center of Kidney and Urology Diseases, Xinqiao Hospital Army Medical University (Third Military Medical University) Chongqing China; ^2^ Department of Gastrointestinal Surgery and Department of Clinical Nutrition, Beijing Shijitan Hospital, Capital Medical University Beijing China; ^3^ National Clinical Research Center for Geriatric Diseases, Xuanwu Hospital, Capital Medical University Beijing China; ^4^ Key Laboratory of Cancer FSMP for State Market Regulation Beijing China; ^5^ Institute for Emergency and Disaster Medicine, Sichuan Provincial People's Hospital, School of Medicine, University of Electronic Science and Technology of China Chengdu China

**Keywords:** chronic kidney disease, GLIS, global leadership initiative in sarcopenia, muscle‐specific strength, sarcopenia

## Abstract

**Background:**

While the Global Leadership Initiative on Sarcopenia (GLIS) is promising to standardize sarcopenia diagnosis, its operational implementation remains largely undefined. This study aims to operationalize GLIS and evaluate its feasibility, diagnostic concordance and clinical relevance.

**Methods:**

This three‐stage, multicenter study enrolled 12 116 participants for cut‐off development (mean age 58.7 years, 48.2% men) and 11 241 participants for outcome analysis (mean age 58.4 years, 49.4% men) from a national survey in China. Another 504 patients with chronic kidney disease were included for validation. We proposed the lower limb skeletal muscle mass to five‐time chair stand test ratio (LFR) to assess muscle‐specific strength (MSS). The GLIS conceptual framework was instantiated into six diagnostic criteria combinations using handgrip strength (HGS), appendicular skeletal muscle mass index (ASMI, estimated using a validated formula) and MSS: (1) all three criteria being low (HAM); (2) low HGS plus low ASMI (HA); (3) low MSS (M); (4) low HGS plus low ASMI, or low MSS (HA/M); (5) low HGS or low MSS (H/M); and (6) low ASMI or low MSS (A/M). Intercriteria concordance of these definitions, relevance with functional outcomes and their concordance with the Asian Working Group for Sarcopenia 2019 (AWGS) criteria were evaluated.

**Results:**

Low MSS cut‐offs were established as < 0.74 for men and < 0.47 for women. Sarcopenia prevalence varied significantly across different definitions: 1055 (8.7%, AWGS), 405 (3.3%, HAM), 619 (5.1%, HA), 2409 (19.9%, M), 2623 (21.6%, HA/M), 3184 (26.3%, H/M) and 3868 (31.9%, A/M). The HA method showed the highest concordance with the AWGS (accuracy = 0.964, *κ* = 0.722, sensitivity = 1.000, specificity = 0.962). The H/M method demonstrated the strongest correlation with functional outcomes and optimal diagnostic performance (AUCs range from 0.566 to 0.729), with superior discrimination for impaired activities of daily living (ADL), other functional measures and global functional scores (*p* < 0.05). All methods independently predicted poor functional outcomes. External validation in CKD showed that the H/M method was either superior or comparable to other methods in identifying disabilities (e.g., predicting functional measures, AUC = 0.627, 95% CI = 0.582–0.672).

**Conclusions:**

This study establishes an operational framework for GLIS using nationally representative data from China and validates its effectiveness in a clinical setting. LFR proves to be a feasible method for assessing MSS. The H/M method effectively captures functional impairment, which may serve as a useful approach for diagnosing sarcopenia. These findings provide actionable benchmarks for sarcopenia research and clinical practice, potentially informing more refined prevention and intervention strategies.

## 1 Introduction

Sarcopenia, a clinically significant condition characterized by the age‐related loss of muscle mass and strength/function [1], has grave health consequences including impaired quality of life, disability, falls, cardiovascular events and increased mortality [1, 2, 3, 4]. Its reported prevalence ranges from 5.5% to 25.7% [5]. However, due to insufficient clinical awareness [6] and limited pharmacological treatment options [7], the true burden of sarcopenia remains largely underestimated [8]. Projections indicate this burden may reach 200 million cases globally by 2050 [9, 10], highlighting an urgent and substantial public health challenge requiring immediate attention [11].

A significant step forward occurred in 2016 when sarcopenia received a specific diagnostic code (M62.84) in the International Classification of Diseases, Tenth Revision, Clinical Modification (ICD‐10‐CM), formally recognizing it as a distinct disease entity [12]. Despite this advance, diagnostic criteria remain inconsistent across institutions, regions and countries [5, 13, 14, 15, 16, 17], and no single set of criteria has achieved global consensus [1, 5, 13]. This lack of a universal diagnostic framework hinders the implementation of standardized clinical management pathways and the aggregation of research findings [1].

To harmonize competing definitions and establish a diagnostic gold standard for sarcopenia, the Global Leadership Initiative in Sarcopenia (GLIS) was recently proposed [1]. GLIS integrates the latest evidence and international expert consensus, which demonstrates the potential to standardize global sarcopenia diagnosis [1]. However, as GLIS is currently a conceptual framework, several challenges impede its application in clinical practice and research. These include the following: (1) identifying the most appropriate indicator for assessing muscle‐specific strength (MSS), a new component of sarcopenia proposed in GLIS; (2) determining sex‐specific MSS cut‐offs; (3) evaluating the diagnostic accuracy of GLIS compared to previous frameworks; and (4) selecting the optimal combination of diagnostic criteria. The GLIS team acknowledges these as significant concerns to be addressed in future work [1].

To address these knowledge gaps, we present an operational diagnostic framework for implementing GLIS. Specifically, we propose a novel metric for assessing MSS and establish sex‐specific thresholds. Furthermore, we compare the diagnostic accuracy of various criteria combinations and assess their association with functional outcomes. Finally, we validated this operational framework in a clinical setting. These findings are crucial for informing strategies for the prevention, diagnosis, surveillance and intervention of sarcopenia and are intended to support global researchers and clinicians in adopting the GLIS framework.

## 2 Methods

### 2.1 Study Design and Population

This was a three‐stage, multicenter cross‐sectional study. We used data from the China Health and Retirement Longitudinal Study (CHARLS), an ongoing nationally representative longitudinal survey focused on ageing‐related health and socioeconomic status in China [18]. We also included 504 patients with chronic kidney disease (CKD) from our institution for external validation. In 2011, CHARLS recruited participants from 10 257 households located in 150 counties/districts and 450 villages or urban communities across 28 provinces using multistage stratified probability‐proportionate‐to‐size sampling in China. Using structured questionnaires and in‐person interviews, high‐quality data were collected from a nationally representative sample of Chinese adults aged ≥ 45 years, encompassing sociodemographic, lifestyle, and health‐related information. A total of 17 708 individuals completed the baseline survey in 2011. Subsequently, follow‐up surveys were conducted every 2 years, with data weighted to ensure population representativeness. Participants were interviewed at their usual residence by interviewers trained by CHARLS staff members at Peking University, Beijing, China. Detailed descriptions of the study design, methods and response rates have been previously described [19].

Participant selection employed a three‐stage process designed to ensure sufficient statistical power for distinct study objectives. Stage 1 included individuals with complete data on age, sex, body height, body weight, handgrip strength (HGS) and the five‐time chair stand test (CST). Stage 2 further restricted inclusion to participants with available functional capacity (FC) data, encompassing activities of daily living (ADL), instrumental activities of daily living (IADL) and other FC measures. Stage 3 was conducted in a clinical setting, including patients who were diagnosed with CKD and received treatment at our institution between March 2022 and July 2025. Exclusion criteria included missing data on study variables or the presence of outlier values. This yielded final samples of 12 116 participants for cut‐off development (Population 1), 11 241 participants for outcome study (Population 2) and 504 patients for external validation (Population 3). A flow chart detailing participant inclusion is presented in Figure S1. The CHARLS research protocol was approved by the Biomedical Ethics Committee of Peking University (approval number: IRB00001052‐11015). All participants, or their legal representatives, signed written informed consent forms to participate in the survey. All patients in Population 3 signed written informed consent forms allowing the use of their data for scientific purposes. All data were analysed anonymously and this study followed the Strengthening the Reporting of Observational Studies in Epidemiology guidelines.

### 2.2 Data Handling

Age (years), sex, body height, body weight, HGS, CST and 20 FC items were extracted from the CHARLS database. Data collection in Population 3 followed the same procedures as those used in CHARLS. Body height was measured to the nearest 0.1 cm using a stadiometer and body weight to the nearest 0.1 kg using a digital floor scale. Body mass index (BMI, kg/m^2^) was calculated as weight (kg) divided by height squared (m^2^) and categorized per Chinese guidelines [20] as underweight (< 18.5), normal (18.5 to < 24), overweight (24 to < 28) or obese (≥ 28). HGS (kg) was measured in the dominant hand using a dynamometer (Population 1 and 2, Model: YuejianTM WL‐1000, Nantong Yuejian Physical Measurement Instrument Co. Ltd., Nantong, China; Population 3, Model: EH101, Guangdong Senssun Weighing Apparatus Group Ltd., Guangdong, China) [19]. Participants were instructed to stand with arms relaxed and perform two maximal squeezes; the highest value was recorded. Low HGS was defined as < 28 kg for men and < 18 kg for women [5]. CST performance—time (seconds) to complete five consecutive chair stands—was measured by CHARLS‐trained interviewers using standardized protocols [19]. Participants folded their arms across their chest and performed five full stands/sits at maximal pace without pauses or arm assistance. A completion time ≥ 12 s indicated low physical performance [5]. Appendicular skeletal muscle (ASM, kg) was retrospectively calculated using a validated anthropometric equation for Chinese populations [21]. This equation (incorporating age, sex, weight and height) demonstrated strong agreement with dual‐energy X‐ray absorptiometry (DEXA) [21]. Appendicular skeletal muscle mass index (ASMI, kg/m^2^) was calculated by adjusting ASM for height squared (m^2^). The 20 FC items comprised 6 ADLs (dressing, bathing, eating, bed, toilet and urination), 5 IADLs (money, medication, shopping, meal and housework) and 9 other FC items (jogging 1 km, walking 1 km, walking 100 m, chair, climbing, stooping, lifting 5 kg, picking and arm). Detailed descriptions of the 20 FC items are provided in Table S1. Difficulty in any task scored 1 point (positive); no difficulty scored 0 (negative). Summary FC scores were derived for ADLs, IADLs, other FCs, and all 20 items combined.

### 2.3 Muscle‐Specific Strength

**FIGURE 1:**
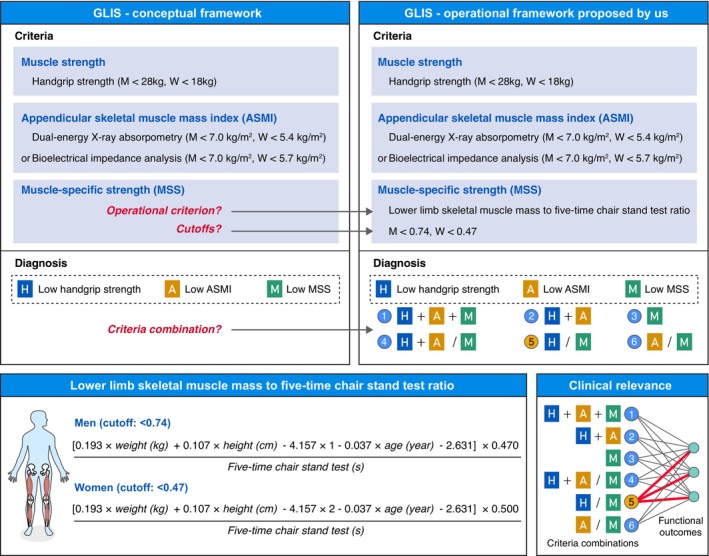
A graphical abstract of the study workflow. ASMI, appendicular skeletal muscle mass index; GLIS, the Global Leadership Initiative in Sarcopenia; M, men; MSS, muscle‐specific strength; W, women.

MSS is a new criterion included in the GLIS framework, defined as muscle strength standardized to muscle size [1]. In this study, we operationalized MSS using a novel index: The lower limb skeletal muscle mass to five‐time chair stand test ratio. This approach was selected for three primary reasons: (1) CST is a well‐established, simple measure of lower limb muscle strength [22], which has been extensively validated in clinical practice [5, 23]; (2) Both ASM and CST are widely used parameters in existing sarcopenia frameworks, such as the Asian Working Group for Sarcopenia 2019 framework (AWGS) [5]. Utilizing these measures ensures seamless integration with the GLIS framework and leverages pre‐existing research datasets for accelerated GLIS investigations; (3) Lower limb skeletal muscle mass can be easily assessed using widely available technologies such as DEXA and bioelectrical impedance analysis. To further enhance the generalizability of our findings in resource‐limited settings lacking advanced body composition tools, we proposed a formula for calculating lower limb skeletal muscle mass (Figure 1). This formula integrates a validated ASM equation [5, 21] with sex‐specific estimates of lower limb skeletal muscle mass as a proportion of total skeletal muscle mass, derived from Asian population data [24]. The sex‐specific cut‐off for low MSS was defined as the lowest 20% (20th percentile) of MSS values within the cut‐off development population. This approach has been widely used in previous studies to derive cut‐offs for muscle‐related parameters [25, 26].

### 2.4 Diagnosis of Sarcopenia

Sarcopenia was retrospectively diagnosed using both the AWGS 2019 version (as a reference) [5] and the GLIS framework [1]. According to the AWGS, sarcopenia is diagnosed with low ASMI plus low muscle strength and/or reduced physical performance. Positivity for all three criteria indicates severe sarcopenia. For the GLIS framework, we evaluated six diagnostic combinations: (1) low HGS plus low ASMI plus low MSS (HAM); (2) low HGS plus low ASMI (HA); (3) low MSS (M); (4) low HGS plus low ASMI, or low MSS (HA/M); (5) low HGS or low MSS (H/M); and (6) low ASMI or low MSS (A/M).

### 2.5 Statistical Analysis

Continuous variables are reported as means ± standard deviation and compared using the Student's *T*‐test. Categorical variables are expressed as numbers (percentages) and compared using the chi‐squared test. Agreement between AWGS and GLIS diagnoses was assessed using Cohen's Kappa statistic (*κ*: 0.00–0.20 = slight, 0.21–0.40 = fair, 0.41–0.60 = moderate, 0.61–0.80 = substantial, 0.81–1.00 = almost perfect) [27], confusion matrix, accuracy, Matthews correlation coefficient (MCC) [28], sensitivity, specificity, positive predictive value (PPV) and negative predictive value (NPV). The performance of sarcopenia criteria in predicting FC outcomes was evaluated using area under the receiver operating characteristic curve (AUC) with 95% confidence intervals (CI). Delong's Test was employed to compare the differences between correlated AUCs. Associations between sarcopenia criteria and FC outcomes were quantified using the Spearman's correlation coefficient, MCC [28] and multivariable logistic regression (adjusted for age, sex and body mass index). All *p*‐values were two‐sided and considered significant if *p* < 0.05. All statistical analyses were performed using R (version 4.3.1, Foundation for Statistical Computing, Vienna, Austria).

## 3 Results

### 3.1 Overview of the Cut‐Off Development Population

The characteristics of the cut‐off development population are presented in Table 1. A total of 12 116 individuals were included, with a mean age of 58.7 years (range: 45–101 years; 5132 participants aged 60 or older), including 5844 men and 6272 women. Among them, 818 (6.8%) were classified as underweight, 6450 (53.2%) as normal weight, 3507 (28.9%) as overweight and 1341 (11.1%) as obese. The cut‐offs for defining low MSS were determined as < 0.74 for men and < 0.47 for women. The prevalence rates of low HGS, low ASMI, low physical performance and low MSS were 1466 (12.1%), 2625 (21.7%), 3572 (29.5%) and 2409 (19.9%), respectively. The prevalence rates of sarcopenia based on different criteria were 1055 (8.7%, AWGS), 405 (3.3%, HAM), 619 (5.1%, HA), 2409 (19.9%, M), 2623 (21.6%, HA/M), 3184 (26.3%, H/M) and 3868 (31.9%, A/M), respectively. AWGS‐defined sarcopenia is associated with all investigated variables (all *p* < 0.05), except for sex (*p* = 0.067).

**TABLE 1:** Baseline characteristics of the cut‐off development population.

		AWGS 2019‐defined sarcopenia		
Characteristics	Overall (*n* = 12 116)	No (n = 11 061)	Yes (*n* = 1055)	*p*
Age, years	58.7 ± 9.2^a^	57.9 ± 8.7	67.5 ± 9.5	< 0.001
Sex, men	5844 (48.2)^b^	5364 (48.5)	480 (45.5)	0.067
Body height, m	1.6 ± 0.1	1.6 ± 0.1	1.5 ± 0.1	< 0.001
Body weight, kg	58.9 ± 11.7	59.6 ± 11.5	51.6 ± 11.4	< 0.001
Body mass index, kg/m2	23.5 ± 3.9	23.6 ± 3.9	21.6 ± 3.9	< 0.001
Body mass index group				< 0.001
Underweight	818 (6.8)	609 (5.5)	209 (19.8)	
Normal	6450 (53.2)	5855 (52.9)	595 (56.4)	
Overweight	3507 (28.9)	3337 (30.2)	170 (16.1)	
Obese	1341 (11.1)	1260 (11.4)	81 (7.7)	
Handgrip strength, kg	31.8 ± 10.8	33.3 ± 9.9	16.5 ± 7.1	< 0.001
Handgrip strength, low	1466 (12.1)	411 (3.7)	1055 (100.0)	< 0.001
ASMI, kg/m^2^	6.8 ± 1.1	6.8 ± 1.1	6.2 ± 1.2	< 0.001
ASMI, low	2625 (21.7)	2006 (18.1)	619 (58.7)	< 0.001
ASM, kg	17.2 ± 4.2	17.4 ± 4.2	14.9 ± 4.1	< 0.001
Chair stand test, s	10.8 ± 4.4	10.4 ± 3.8	15.3 ± 6.9	< 0.001
Chair stand test, impaired	3572 (29.5)	2794 (25.3)	778 (73.7)	< 0.001
Muscle‐specific strength	1.0 ± 2.8	1.0 ± 2.9	0.5 ± 0.2	< 0.001
Muscle‐specific strength, low	2409 (19.9)	1735 (15.7)	674 (63.9)	< 0.001
AWGS 2019, grade				< 0.001
Not sarcopenia	11 061 (91.3)	11 061 (100.0)	0 (0.0)	
Sarcopenia	713 (5.9)	0 (0.0)	713 (67.6)	
Severe sarcopenia	342 (2.8)	0 (0.0)	342 (32.4)	
AWGS 2019, yes	1055 (8.7)	0 (0.0)	1055 (100.0)	<0.001
GLIS HAM, yes	405 (3.3)	0 (0.0)	405 (38.4)	<0.001
GLIS HA, yes	619 (5.1)	0 (0.0)	619 (58.7)	<0.001
GLIS M, yes	2409 (19.9)	1735 (15.7)	674 (63.9)	<0.001
GLIS HA/M, yes	2623 (21.6)	1735 (15.7)	888 (84.2)	<0.001
GLIS H/M, yes	3184 (26.3)	2129 (19.2)	1055 (100.0)	<0.001
GLIS A/M, yes	3868 (31.9)	2980 (26.9)	888 (84.2)	<0.001

Abbreviations: AWGS 2019, the Asian Working Group for Sarcopenia 2019 framework; ASMI, appendicular skeletal muscle mass index; ASM, appendicular skeletal muscle mass; GLIS, the Global Leadership Initiative in Sarcopenia; H/A/M, low handgrip strength, low appendicular skeletal muscle mass index and low muscle‐specific strength.

^a^ Mean ± standard deviation, all such values.

^b^ Number (percentage), all such values.

### 3.2 Between‐Criteria Analysis

**FIGURE 2:**
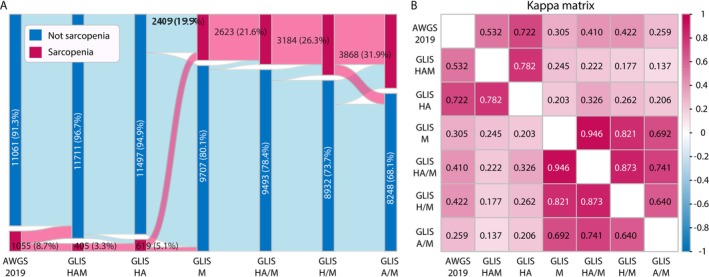
Prevalence and concordance of different criteria for sarcopenia. AWGS 2019, the Asian Working Group for Sarcopenia 2019 framework; GLIS, the Global Leadership Initiative in Sarcopenia; HAM, low handgrip strength, low appendicular skeletal muscle mass index and low muscle‐specific strength. (A) Prevalence and percentage of sarcopenia by different diagnostic criteria. (B) Kappa matrix of the concordance between different diagnostic criteria.

Changes in the numbers and proportions for sarcopenia across different criteria are shown in Figure 2A. Overall, diagnostic criteria significantly impact the prevalence of sarcopenia, ranging from 3.3% (HAM) to 31.9% (A/M). Between‐criteria concordance is analysed using a Kappa matrix (Figure 2B) and a confusion matrix (Table S2). Using AWGS as the reference standard, only HA demonstrated substantial concordance (*κ* = 0.722). The remaining criteria showed moderate concordance (HAM: *κ* = 0.532; HA/M: *κ* = 0.410; H/M: *κ* = 0.422) or fair concordance (M: *κ* = 0.305; A/M: *κ* = 0.259). Among the GLIS criteria themselves, substantial concordance (*κ* > 0.6) was observed between M, HA, H/M and A/M. Concordance between all other GLIS criteria pairs was lower (*κ* < 0.6).

**FIGURE 3:**
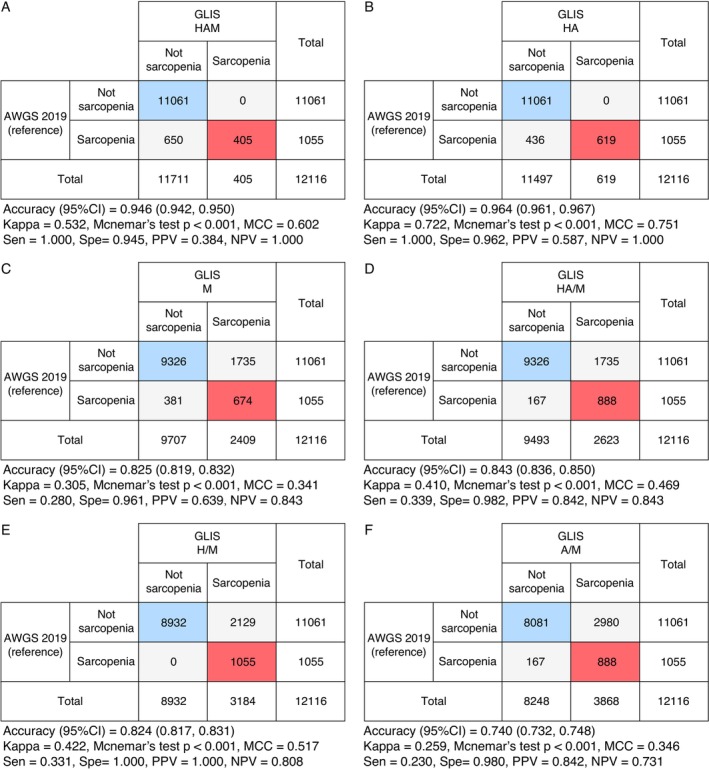
Accuracy of the Global Leadership Initiative in Sarcopenia (GLIS) criteria referenced to the Asian Working Group for Sarcopenia (AWGS) 2019 framework. H/A/M, low handgrip strength, low appendicular skeletal muscle mass index, and low muscle‐specific strength; MCC, Matthews Correlation Coefficient; NPV, negative predictive value; PPV, positive predictive value; Sen, sensitivity; Spe, specificity. (A) GLIS HAM versus AWGS 2019. (B) GLIS HA versus AWGS 2019. (C) GLIS M versus AWGS 2019. (D) GLIS HA/M versus AWGS 2019. (E) GLIS H/M versus AWGS 2019. (F) GLIS A/M versus AWGS 2019.

Detailed concordance analyses between AWGS and different GLIS criteria are shown in Figure 3A–F. Compared to AWGS, the HA criteria demonstrated the greatest concordance (Accuracy = 0.964, 95% CI = 0.961 to 0.967; *κ* = 0.722; MCC = 0.751; Sensitivity = 1.000; Specificity = 0.962; PPV = 0.587; NPV = 1.000). In contrast, the A/M criteria showed the poorest agreement with AWGS (Accuracy = 0.740, 95% CI = 0.732 to 0.748; *κ* = 0.259; MCC = 0.346; Sensitivity = 0.230; Specificity = 0.980; PPV = 0.842; NPV = 0.731).

### 3.3 Overview of the Outcome Study Population

The characteristics of the outcome study population (*n* = 11 241) are presented in Table 2 Distributions of study variables and their associations with AWGS‐defined sarcopenia were consistent with those observed in the cut‐off development population. Among the 20 FC items, jogging 1 km had the highest positivity rate (*n* = 5386, 47.9%), while the fewest participants reported difficulty with eating (*n* = 130, 1.2%). There are 1416 (12.6%), 1943 (17.3%), 7182 (63.9%) and 7431 (66.1%) participants with at least one positive FC item for the ADL, IADL, other FC and summary scores, respectively. AWGS‐defined sarcopenia was significantly associated with positive responses across all 20 FC items and all four composite scores (all *p* < 0.05).

**TABLE 2:** Baseline characteristics of the outcome study population.

		AWGS 2019‐defined sarcopenia		
*n*	Overall (*n* = 11 241)	No (*n* = 10 345)	Yes (*n* = 896)	*p*
Age, years	58.4 ± 9.0^a^	57.6 ± 8.6	67.1 ± 9.4	< 0.001
Sex, men	5551 (49.4)^b^	5126 (49.6)	425 (47.4)	0.237
Body height, m	1.6 ± 0.1	1.6 ± 0.1	1.5 ± 0.1	< 0.001
Body weight, kg	59.0 ± 11.7	59.7 ± 11.5	51.4 ± 11.2	< 0.001
Body mass index, kg/m^2^	23.4 ± 3.9	23.6 ± 3.8	21.4 ± 3.8	< 0.001
Body mass index group				< 0.001
Underweight	744 (6.6)	555 (5.4)	189 (21.1)	
Normal	6029 (53.6)	5524 (53.4)	505 (56.4)	
Overweight	3254 (28.9)	3117 (30.1)	137 (15.3)	
Obese	1214 (10.8)	1149 (11.1)	65 (7.3)	
Handgrip strength, kg	32.2 ± 10.7	33.6 ± 9.9	16.7 ± 7.3	< 0.001
Handgrip strength, low	1277 (11.4)	381 (3.7)	896 (100.0)	< 0.001
ASMI, kg/m^2^	6.8 ± 1.1	6.8 ± 1.1	6.2 ± 1.2	< 0.001
ASMI, low	2384 (21.2)	1842 (17.8)	542 (60.5)	< 0.001
ASM, kg	17.3 ± 4.2	17.5 ± 4.2	15.0 ± 4.0	< 0.001
Chair stand test, s	10.5 ± 4.0	10.2 ± 3.6	14.6 ± 5.9	< 0.001
Chair stand test, impaired	3115 (27.7)	2475 (23.9)	640 (71.4)	< 0.001
Muscle‐specific strength	1.0 ± 2.9	1.0 ± 3.0	0.5 ± 0.2	< 0.001
Muscle‐specific strength, low	2070 (18.4)	1513 (14.6)	557 (62.2)	< 0.001
AWGS 2019, grade				< 0.001
Not sarcopenia	10 345 (92.0)	10 345 (100.0)	0 (0.0)	
Sarcopenia	610 (5.4)	0 (0.0)	610 (68.1)	
Severe sarcopenia	286 (2.5)	0 (0.0)	286 (31.9)	
AWGS 2019, yes	896 (8.0)	0 (0.0)	896 (100.0)	< 0.001
GLIS HAM, yes	345 (3.1)	0 (0.0)	345 (38.5)	< 0.001
GLIS HA, yes	542 (4.8)	0 (0.0)	542 (60.5)	< 0.001
GLIS M, yes	2070 (18.4)	1513 (14.6)	557 (62.2)	< 0.001
GLIS HA/M, yes	2267 (20.2)	1513 (14.6)	754 (84.2)	< 0.001
GLIS H/M, yes	2774 (24.7)	1878 (18.2)	896 (100.0)	< 0.001
GLIS A/M, yes	3444 (30.6)	2690 (26.0)	754 (84.2)	< 0.001
Functional capacity				
ADL all, continuous	0.2 ± 0.7	0.2 ± 0.6	0.6 ± 1.2	< 0.001
ADL all, ≥ 1	1416 (12.6)	1165 (11.3)	251 (28.0)	< 0.001
Dressing	320 (2.8)	237 (2.3)	83 (9.3)	< 0.001
Bathing	356 (3.2)	266 (2.6)	90 (10.0)	< 0.001
Eating	130 (1.2)	80 (0.8)	50 (5.6)	< 0.001
Bed	309 (2.7)	244 (2.4)	65 (7.3)	< 0.001
Toilet	938 (8.3)	766 (7.4)	172 (19.2)	< 0.001
Urination	331 (2.9)	273 (2.6)	58 (6.5)	< 0.001
IADL all, continuous	0.3 ± 0.8	0.3 ± 0.7	0.7 ± 1.3	< 0.001
IADL all, ≥ 1	1943 (17.3)	1642 (15.9)	301 (33.6)	< 0.001
Money	1129 (10.0)	938 (9.1)	191 (21.3)	< 0.001
Medication	549 (4.9)	454 (4.4)	95 (10.6)	< 0.001
Shopping	605 (5.4)	471 (4.6)	134 (15.0)	< 0.001
Meal	532 (4.7)	414 (4.0)	118 (13.2)	< 0.001
Housework	588 (5.2)	455 (4.4)	133 (14.8)	< 0.001
Other FC items, continuous	1.6 ± 1.7	1.5 ± 1.7	2.8 ± 2.1	< 0.001
Other FC items, ≥ 1	7182 (63.9)	6419 (62.0)	763 (85.2)	< 0.001
Jogging 1 km	5386 (47.9)	4736 (45.8)	650 (72.5)	< 0.001
Walking 1 km	923 (8.2)	733 (7.1)	190 (21.2)	< 0.001
Walking 100 m	157 (1.4)	114 (1.1)	43 (4.8)	< 0.001
Chair	2617 (23.3)	2265 (21.9)	352 (39.3)	< 0.001
Climbing	4069 (36.2)	3562 (34.4)	507 (56.6)	< 0.001
Stooping	2866 (25.5)	2502 (24.2)	364 (40.6)	< 0.001
Lifting 5 kg	838 (7.5)	643 (6.2)	195 (21.8)	< 0.001
Picking	261 (2.3)	208 (2.0)	53 (5.9)	< 0.001
Arm	868 (7.7)	718 (6.9)	150 (16.7)	< 0.001
Summary all, continuous	2.1 ± 2.6	1.9 ± 2.4	4.1 ± 3.8	< 0.001
Summary all, ≥ 1	7431 (66.1)	6652 (64.3)	779 (86.9)	< 0.001

Abbreviations: ADL, Activities of Daily Living; ASM, appendicular skeletal muscle mass; ASMI, appendicular skeletal muscle mass index; AWGS 2019, the Asian Working Group for Sarcopenia 2019 framework; FC, functional capacity; GLIS, the Global Leadership Initiative in Sarcopenia; H/A/M, low handgrip strength, low appendicular skeletal muscle mass index, and low muscle‐specific strength; IADL, Instrumental Activities of Daily Living.

^a^ Mean ± standard deviation, all such values.

^b^ Number (percentage), all such values.

### 3.4 Clinical Relevance

**FIGURE 4:**
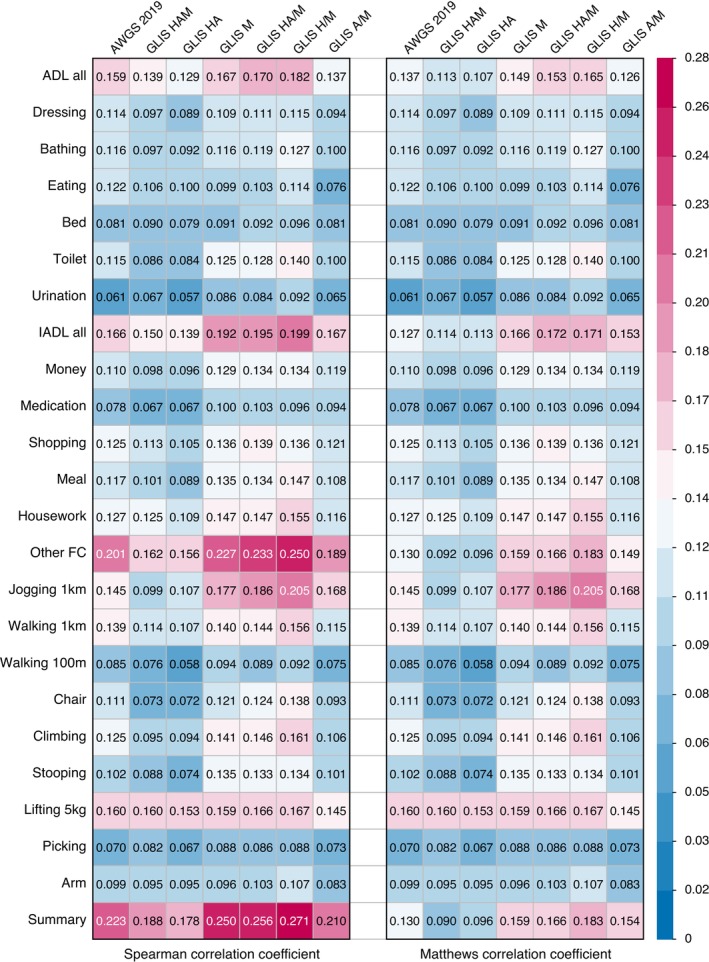
Association between different diagnostic criteria of sarcopenia and functional capacity (FC) outcomes. ADL, activities of daily living; AWGS 2019, the Asian Working Group for Sarcopenia 2019 framework; GLIS, the Global Leadership Initiative in Sarcopenia; HAM, low handgrip strength, low appendicular skeletal muscle mass index and low muscle‐specific strength; IADL, instrumental activities of daily living.

Correlations between different diagnostic criteria and functional capacity items are visualized in Figure 4. Among the seven diagnostic criteria evaluated, the H/M method exhibited the strongest clinical relevance. It demonstrated the highest Spearman's correlation coefficients with 15 of 20 FC indicators (excluding eating, medication, shopping, walking 100 m and stooping) and all four summary scores. These findings were consistent when assessed using the MCC.

The comparison results for different diagnostic criteria in relation to FC items are shown in Table S3. The H/M method again demonstrated the highest AUC (95% CI) for predicting all 20 FC items and four summary scores, with AUCs ranging from 0.566 (95% CI: [0.556–0.576]) for stooping to 0.729 (95% CI: [0.690–0.769]) for eating. We evaluated the statistical significance of the H/M method's superiority using Delong's test. Of 144 pairwise comparisons (6 criteria comparisons × 24 outcomes), 18 tests showed no significant difference (all *p* > 0.05). For the ADL, other FC and summary scores, the H/M method significantly outperformed all six alternative criteria (all *p* < 0.05). For IADL, it surpassed five methods (all *p* < 0.05) but showed comparable performance to the A/M method (*p* = 0.458).

Multivariable logistic regression results (Table S4) confirmed that all diagnostic criteria remained significantly associated with increased odds of all FC outcomes after adjusting for age, sex, and BMI. For example, participants diagnosed with sarcopenia using the H/M method had a nearly sixfold increase in odds of eating‐related limitations compared to those without sarcopenia (OR = 5.98; 95% CI: 3.96–9.03).

### 3.5 External Validation

The characteristics of the external validation population (*n* = 504) are presented in Table S5. The mean age of this population was 48.2 years, including 247 men and 257 women. A total of 213 (42.3%) patients were defined as having sarcopenia based on the H/M method. The distributions of study variables and their associations with GLIS H/M‐defined sarcopenia were generally consistent with those observed in the other two populations. GLIS H/M‐defined sarcopenia was associated with positive responses across 12 FC items and three composite scores (all *p* < 0.05).

The comparison results for different diagnostic criteria in relation to four composite FC scores are shown in Table S6. The H/M method demonstrated apparently the highest AUC (95% CI) for predicting all four composite scores, with AUCs ranging from 0.549 (95% CI: [0.487–0.611]) for ADL to 0.627 (95% CI: [0.582–0.672]) for other FC. Subsequent Delong's tests showed that the H/M method was either superior to or comparable with other methods in identifying FC‐represented disabilities in patients. Multivariable logistic regression results (Table S7) showed that the H/M method‐defined sarcopenia was associated with increased odds of impairment across all four composite FC scores.

## 4 Discussion

This large‐scale study utilized data from both a multicenter, nationally representative survey and a kidney disease centre that employed standardized data collection. We addressed several key knowledge gaps in the latest GLIS conceptual framework. We proposed a novel index for assessing MSS: the lower limb ASM to CST ratio. Derived from widely accepted parameters, this index should bridge prior frameworks to GLIS criteria and may facilitate reanalysis of existing datasets in future studies. Furthermore, using a representative sample, we established sex‐specific MSS cut‐off values and evaluated multiple GLIS diagnostic criteria combinations, assessing their accuracy and correlations with functional outcomes. These findings provide actionable benchmarks for sarcopenia research and inform future prevention/intervention strategies in both public health and clinical scenarios.

In this study, we observed a slightly higher correlation between GLIS‐defined sarcopenia and IADL than with basic ADL (Figure 4). This aligns with a prospective cohort study showing EWGSOP‐defined sarcopenia increased IADL disability risk by 2.3‐fold over 4 years, though no consistent ADL association was observed [29]. Similarly, studies report higher IADL limitations in older women with sarcopenia, with inconsistent ADL findings in men [30]. A review conducted by the GLIS group also noted moderate IADL disability risk linkage but inconclusive ADL evidence [4]. Conversely, hospitalized populations exhibit sarcopenia‐ADL associations without IADL correlations [31]. While GLIS adopts both ADL and IADL as clinical outcomes, current evidence on sarcopenia‐ADL/IADL relationships remains inconsistent [4, 29, 30, 31]. Discrepancies may stem from (1) heterogeneous sarcopenia definitions, (2) variable ADL/IADL assessment methodologies and (3) population differences. Given that there are still relatively few studies based on the GLIS framework, more evidence is needed to draw definitive conclusions. Nevertheless, the unique value of our study lies in its re‐evaluation of this relationship using both GLIS and former AWGS criteria, enabling comparative analysis and providing critical references for future research.

MSS is not a new concept in sarcopenia research [32], but it is a core component introduced in the GLIS framework [1]. Notably, MSS had the lowest agreement (80.8%) among all statements finally accepted in the GLIS Delphi process, marginally exceeding the predefined acceptance threshold (> 80%) [1]. This reflects a certain degree of disagreement among experts in the GLIS group regarding the role of MSS in sarcopenia diagnostics. This divergence may stem from two significant factors: first, the absence of a clinically feasible, reliable and universally accepted MSS assessment tool [1, 32]; second, limited evidence that MSS inclusion enhances the clinical relevance of sarcopenia diagnosis [4]. Our study addresses both concerns: the proposed MSS index comprises simple, evidence‐based and widely accessible components, permitting implementation in nearly all clinical settings. Crucially, sarcopenia defined solely by MSS demonstrated stronger correlations with ADL, IADL, other FC items and summary FC scores than AWGS‐defined sarcopenia (Figure 4). These findings collectively support that integrating MSS into the GLIS diagnostic framework could augment its clinical utility.

A pertinent question is why HGS and upper‐limb muscle mass were excluded from our MSS assessment. In an exploratory analysis, we used HGS standardized to arm skeletal muscle to define MSS and to implement the GLIS framework. However, this approach showed weaker correlations across 20 FC outcomes than even the AWGS 2019 criteria (data not shown). This aligns with established literature: Prevailing objective measures of physical performance (e.g., usual gait speed, 6‐min walk test, stair‐climb power test, timed‐up‐and‐go test, CST and Short Physical Performance Battery) predominantly evaluate lower limb musculoskeletal system [5, 14, 33, 34, 35]. Lower limb strength is inherently prioritized in functional assessments due to its direct association with an individual's mobility. Another study found that, contrary to the expected decline in maximal oxygen consumption with age, older individuals exhibit an increased metabolic capacity in the forearm during handgrip exercise, allowing them to achieve comparable maximum work rates to younger individuals [36]. This suggests that the upper extremities may age differently in terms of physiology and muscle volume of the forearm flexors compared to the locomotor muscles of the lower limbs. These observations may help explain our exploratory results and further validate the superiority of our lower limb‐focused MSS index.

The uniqueness of this study lies in several key aspects. First, it utilizes data from a multicenter, nationally representative survey with standardized collection protocols. Second, China has the world's largest elderly population, and this study addresses a prevalent ageing‐related issue of global interdisciplinary significance. Our findings, therefore, have substantial implications for policymakers that are planning appropriate surveillance and intervention strategies for an ageing population. They may also directly inform clinical guidelines while advancing evidence‐based diagnosis and interventions worldwide. Third, our sample derived from a survey that includes both community‐dwelling healthy adults and those with chronic diseases. We also employed a clinical sample for external validation. This should enhance the potential generalizability of our results to both public health and clinical settings.

There are several potential limitations and generalizability issues in this study that must be noted. First, Asian populations may have anthropometric and physical differences compared to other ethnic or geographic groups [5, 13]. Consequently, these findings necessitate re‐evaluation in non‐Asian populations, particularly concerning the sex‐specific cut‐offs for MSS derived from Asian populations. A key issue is the calculation of lower limb skeletal muscle mass using large‐scale data from other populations, such as Caucasian individuals. Potential data sources may include the UK Biobank and the National Health and Nutrition Examination Survey. Second, we estimated ASM using an anthropometric equation [21]. While DEXA or BIA might offer greater precision, this equation demonstrates strong concordance with DEXA [21] and aligns with methodologies widely adopted in prior research [5, 37]. Its pragmatic utility also enhances the feasibility of implementing the GLIS in resource‐limited settings. Nevertheless, future studies should employ more advanced ASM assessment tools to validate our results. Future studies could evaluate whether other approaches, such as estimating leg muscle volume through anthropometric methods [38], can provide a more robust estimation of lower limb muscle mass. Third, the cut‐off development for MSS was conducted in a population aged 45 to 101 years. Therefore, the generalizability to other age groups requires further assessment. Fourth, sarcopenia diagnoses were derived retrospectively, introducing possible misclassification bias. We mitigated this risk by excluding participants with incomplete data, and prior studies support the feasibility of this approach [26, 37]. Prospective longitudinal studies remain essential to verify our findings—particularly the association between GLIS‐defined sarcopenia and FC trajectories. Finally, direct strength measurements of the lower limb muscles (such as the isometric knee extension test [39]), combined with localized anthropometric estimates of muscle mass in the lower limbs, may enhance the clinical precision of MSS and GLIS‐based diagnoses of sarcopenia. Future research could employ these methods to replicate our findings. Addressing these limitations will strengthen future research.

In conclusion, we developed and validated an operational GLIS framework, establishing a novel index for MSS with sex‐specific thresholds using data from a national survey. Critically, low HGS or low MSS within the GLIS framework optimally identifies outcome‐related sarcopenia. These findings may advance the scientific basis for adopting GLIS and inform targeted sarcopenia prevention and intervention strategies.

## Author Contributions

Liangyu Yin contributed to the conception and design of the research. Liangyu Yin, Jinghong Zhao, Yu Cao and Mengda Tang contributed to the acquisition of the data. Liangyu Yin, Hanping Shi and Hua Jiang contributed to the analysis of the data. All authors contributed to the interpretation of the data. Liangyu Yin drafted the manuscript; and all authors critically revised the manuscript, agree to be fully accountable for ensuring the integrity and accuracy of the work, and read and approved the final manuscript.

## Funding

This work was supported by the Key Program of the Joint Funds of the National Natural Science Foundation of China (U22A20279), the National Natural Science Foundation of China (82304131), the Natural Science Foundation of Chongqing, China (CSTB2024NSCQ‐MSX1233), the China Postdoctoral Science Foundation (2025M784537) and the Key Project of Chongqing Technology Development and Application Program (CSTB2023TIAD‐KPX0060).

## Ethics Statement

Authors certify that the ethical guidelines for publishing of the *Journal of Cachexia, Sarcopenia and Muscle:* update 2019 has been followed [40]. National and international research ethics guidelines were followed, including the Deontological Code of Ethics and the 1964 Declaration of Helsinki and its later amendments. All patients provide written consent for the use of their data, and the study protocol of CHARLS was approved by the Ethical Review Committee of Peking University (approval number: IRB00001052‐11015).

## Conflicts of Interest

The authors declare no conflicts of interest.

## Supporting information


**Table S1:** Functional capacity items included for analysis in the present study.
**Table S2:** Confusion matrix of all criteria investigated for diagnosing sarcopenia in the cut‐off development population.
**Table S3:** Performance of different sarcopenia criteria for diagnosing functional capacity outcomes in the outcome study population.
**Table S4:** Associations of different sarcopenia criteria with functional capacity in the outcome study population.
**Table S5:** Baseline characteristics of the external validation population.
**Table S6:** Performance of different sarcopenia criteria for diagnosing functional capacity outcomes in the external validation population.
**Table S7:** Associations of different sarcopenia criteria with functional capacity in the external validation population.
**Figure S1:** A flow chart of the subject inclusion. ADL, activities of daily living; CHARLS, the China Health and Retirement Longitudinal Study; CKD, chronic kidney disease; FC, functional capacity; IADL, instrumental activities of daily living.
