# Supplementary material for: Operationalizing the Global Leadership Initiative in Sarcopenia: Muscle‐Specific Strength, Optimal Criteria and Clinical Relevance
Source: J Cachexia Sarcopenia Muscle. 2026 Jan 30;17(1):e70222. doi: 10.1002/jcsm.70222 (PMC12858665; doi:10.1002/jcsm.70222)
Supplement: Supplementary file 1 — Table S1: Functional capacity items included for analysis in the present study. Table S2: Confusion matrix of all criteria investigated for diagnosing sarcopenia in the cut‐off development population. Table S3: Performance of different sarcopenia criteria for diagnosing functional capacity outcomes in the outcome study population. Table S4: Associations of different sarcopenia criteria with functional capacity in the outcome study population. Table S5: Baseline characteristics of the external validation population. Table S6: Performance of different sarcopenia criteria for diagnosing functional capacity outcomes in the external validation population. Table S7: Associations of different sarcopenia criteria with functional capacity in the external validation population. Figure S1: A flow chart of the subject inclusion. ADL, activities of daily living; CHARLS, the China Health and Retirement Longitudinal Study; CKD, chronic kidney disease; FC, functional capacity; IADL, instrumental activities of daily living. [file JCSM-17-e70222-s001.pdf]

Supplementary files

Number	Description
1	**Table S1.** Functional capacity items included for analysis in the present study
2	**Table S2.** Confusion matrix of all criteria investigated for diagnosing sarcopenia in the cutoff development population
3	**Table S3.** Performance of different sarcopenia criteria for diagnosing functional capacity outcomes in the outcome study population
4	**Table S4.** Associations of different sarcopenia criteria with functional capacity in the outcome study population
5	**Table S5.** Baseline characteristics of the external validation population
6	**Table S6.** Performance of different sarcopenia criteria for diagnosing functional capacity outcomes in the external validation population
7	**Table S7.** Associations of different sarcopenia criteria with functional capacity in the external validation population
8	**Figure S1.** A flowchart of the subject inclusion. CHARLS, the China Health and Retirement Longitudinal Study; ADL, activities of daily living; IADL, instrumental activities of daily living; FC, functional capacity; CKD, chronic kidney disease

Table S1. Functional capacity indices included for analysis in the present study

Category	Index	Detailed description
ADL	Dressing	Do you have some difficulty with dressing?
	Bathing	Do you have some difficulty with bathing?
	Eating	Do you have some difficulty with eating?
	Bed	Do you have some difficulty with getting in and out of bed?
	Toilet	Do you have some difficulty with using the toilet?
	Urination	Do you have some difficulty with controlling urination and defecation?
IADL	Money	Do you have some difficulty with managing money?
	Medication	Do you have some difficulty with taking medications?
	Shopping	Do you have some difficulty with shopping for groceries?
	Meal	Do you have some difficulty with preparing meals?
	Housework	Do you have some difficulty with cleaning house?
Other	Jogging 1km	Do you have some difficulty with running or jogging 1km?
	Walking 1km	Do you have some difficulty with walking 1km?
	Walking 100m	Do you have some difficulty with walking 100m?
	Chair	Do you have some difficulty with getting up from a chair after sitting for long periods?
	Climbing	Do you have some difficulty with climbing several flights of stairs without resting?
	Stooping	Do you have some difficulty with stooping, kneeling, or crouching?
	Lifting 5kg	Do you have some difficulty with lifting or carrying weights over 5kg?
	Picking	Do you have some difficulty with picking up a coin from the table?
	Arm	Do you have some difficulty with reaching arms above shoulder level?

Abbreviations: ADL, activities of daily living; IADL, instrumental activities of daily living.

Table S2. Confusion matrix of all criteria investigated for diagnosing sarcopenia in the cutoff development population

	Criteria	AWGS		GLIS HAM		GLIS HA		GLIS M		GLIS HA/M		GLIM H/M		GLIS A/M	
Criteria	Diagnosis	Not SP	SP	Not SP	SP	Not SP	SP	Not SP	SP	Not SP	SP	Not SP	SP	Not SP	SP
AWGS	Not SP	11061	0	11061	0	11061	0	9326	1735	9326	1735	8932	2129	8081	2980
	SP	0	1055	650	405	436	619	381	674	167	888	0	1055	167	888
GLIS HAM	Not SP	11061	650	11711	0	11497	214	9707	2004	9493	2218	8932	2779	8248	3463
	SP	0	405	0	405	0	405	0	405	0	405	0	405	0	405
GLIS HA	Not SP	11061	436	11497	0	11497	0	9493	2004	9493	2004	8932	2565	8248	3249
	SP	0	619	214	405	0	619	214	405	0	619	0	619	0	619
GLIS M	Not SP	9326	381	9707	0	9493	214	9707	0	9493	214	8932	775	8248	1459
	SP	1735	674	2004	405	2004	405	0	2409	0	2409	0	2409	0	2409
GLIS HA/M	Not SP	9326	167	9493	0	9493	0	9493	0	9493	0	8932	561	8248	1245
	SP	1735	888	2218	405	2004	619	214	2409	0	2623	0	2623	0	2623
GLIM H/M	Not SP	8932	0	8932	0	8932	0	8932	0	8932	0	8932	0	7687	1245
	SP	2129	1055	2779	405	2565	619	775	2409	561	2623	0	3184	561	2623
GLIS A/M	Not SP	8081	167	8248	0	8248	0	8248	0	8248	0	7687	561	8248	0
	SP	2980	888	3463	405	3249	619	1459	2409	1245	2623	1245	2623	0	3868

Abbreviations: AWGS 2019, the Asian Working Group for Sarcopenia 2019 framework; GLIS, the Global Leadership Initiative in Sarcopenia; H/A/M, low handgrip strength, low appendicular skeletal muscle mass index and low muscle-specific strength; SP, sarcopenia.

Table S3. Performance of different sarcopenia criteria for diagnosing functional capacity outcomes in the outcome study population

	AUC (95%CI)							Delong's test (Reference = GLIS H/M)					
	AWGS 2019	GLIS HAM	GLIS HA	GLIS M	GLIS HA/M	GLIS H/M	GLIS A/M	*P1*	*P2*	*P3*	*P4*	*P5*	*P6*
ADL all	0.556 (0.546-0.566)	0.529 (0.522-0.537)	0.535 (0.526-0.543)	0.587 (0.574-0.600)	0.592 (0.579-0.605)	0.607 (0.593-0.620)	0.588 (0.574-0.601)	<0.001	<0.001	<0.001	<0.001	<0.001	0.020
Dressing	0.592 (0.568-0.617)	0.550 (0.532-0.569)	0.557 (0.537-0.577)	0.627 (0.600-0.655)	0.634 (0.607-0.662)	0.650 (0.622-0.677)	0.630 (0.603-0.658)	<0.001	<0.001	<0.001	0.010	0.039	0.098
Bathing	0.589 (0.567-0.612)	0.548 (0.531-0.565)	0.556 (0.537-0.575)	0.628 (0.602-0.654)	0.637 (0.610-0.663)	0.657 (0.631-0.683)	0.632 (0.606-0.658)	<0.001	<0.001	<0.001	0.001	0.007	0.028
Eating	0.654 (0.612-0.696)	0.586 (0.551-0.620)	0.600 (0.563-0.637)	0.679 (0.636-0.722)	0.694 (0.651-0.736)	0.729 (0.690-0.769)	0.664 (0.622-0.706)	<0.001	<0.001	<0.001	0.002	0.012	<0.001
Bed	0.567 (0.544-0.590)	0.547 (0.529-0.566)	0.552 (0.532-0.572)	0.608 (0.581-0.636)	0.613 (0.585-0.640)	0.626 (0.598-0.654)	0.614 (0.585-0.642)	<0.001	<0.001	<0.001	0.037	0.070	0.308
Toilet	0.557 (0.544-0.569)	0.527 (0.518-0.536)	0.532 (0.522-0.543)	0.587 (0.572-0.603)	0.593 (0.577-0.609)	0.609 (0.593-0.625)	0.583 (0.566-0.599)	<0.001	<0.001	<0.001	<0.001	<0.001	<0.001
Urination	0.549 (0.529-0.570)	0.534 (0.518-0.550)	0.536 (0.518-0.553)	0.598 (0.572-0.624)	0.600 (0.573-0.627)	0.617 (0.590-0.644)	0.588 (0.561-0.615)	<0.001	<0.001	<0.001	0.022	0.021	0.009
IADL all	0.545 (0.537-0.554)	0.526 (0.520-0.532)	0.532 (0.525-0.539)	0.585 (0.574-0.596)	0.591 (0.580-0.602)	0.597 (0.586-0.609)	0.594 (0.582-0.606)	<0.001	<0.001	<0.001	<0.001	0.031	0.458
Money	0.550 (0.539-0.561)	0.528 (0.520-0.536)	0.534 (0.525-0.544)	0.583 (0.569-0.597)	0.589 (0.575-0.604)	0.596 (0.581-0.611)	0.591 (0.576-0.606)	<0.001	<0.001	<0.001	0.003	0.055	0.421
Medication	0.549 (0.533-0.565)	0.527 (0.515-0.539)	0.533 (0.520-0.546)	0.590 (0.570-0.610)	0.596 (0.575-0.617)	0.596 (0.575-0.617)	0.600 (0.579-0.622)	<0.001	<0.001	<0.001	0.269	0.960	0.633
Shopping	0.575 (0.558-0.592)	0.543 (0.530-0.556)	0.550 (0.536-0.564)	0.617 (0.597-0.637)	0.623 (0.603-0.643)	0.630 (0.610-0.650)	0.624 (0.603-0.644)	<0.001	<0.001	<0.001	0.025	0.165	0.462
Meal	0.575 (0.557-0.592)	0.541 (0.528-0.554)	0.545 (0.530-0.559)	0.623 (0.602-0.645)	0.627 (0.606-0.648)	0.650 (0.628-0.671)	0.617 (0.596-0.639)	<0.001	<0.001	<0.001	<0.001	<0.001	<0.001
Housework	0.577 (0.560-0.594)	0.548 (0.535-0.562)	0.553 (0.538-0.567)	0.628 (0.608-0.648)	0.632 (0.612-0.653)	0.650 (0.629-0.670)	0.620 (0.599-0.641)	<0.001	<0.001	<0.001	0.001	0.002	0.001
Other FC	0.537 (0.532-0.541)	0.516 (0.514-0.519)	0.521 (0.518-0.525)	0.564 (0.558-0.571)	0.569 (0.562-0.576)	0.582 (0.575-0.590)	0.572 (0.563-0.580)	<0.001	<0.001	<0.001	<0.001	<0.001	0.004
Jogging 1km	0.539 (0.534-0.544)	0.517 (0.514-0.520)	0.523 (0.519-0.527)	0.569 (0.562-0.576)	0.575 (0.567-0.582)	0.589 (0.581-0.596)	0.577 (0.569-0.586)	<0.001	<0.001	<0.001	<0.001	<0.001	0.002
Walking 1km	0.569 (0.555-0.582)	0.536 (0.526-0.545)	0.542 (0.531-0.552)	0.599 (0.583-0.615)	0.605 (0.589-0.621)	0.622 (0.606-0.639)	0.597 (0.580-0.614)	<0.001	<0.001	<0.001	<0.001	<0.001	<0.001
Walking 100m	0.598 (0.563-0.634)	0.555 (0.528-0.583)	0.553 (0.525-0.581)	0.655 (0.616-0.695)	0.653 (0.613-0.692)	0.669 (0.630-0.708)	0.648 (0.610-0.687)	<0.001	<0.001	<0.001	0.240	0.137	0.218
Chair	0.536 (0.529-0.543)	0.515 (0.510-0.519)	0.518 (0.513-0.524)	0.556 (0.546-0.565)	0.559 (0.549-0.568)	0.571 (0.560-0.581)	0.551 (0.540-0.561)	<0.001	<0.001	<0.001	<0.001	<0.001	<0.001
Climbing	0.535 (0.529-0.541)	0.517 (0.513-0.521)	0.521 (0.516-0.525)	0.557 (0.549-0.565)	0.561 (0.553-0.569)	0.572 (0.564-0.581)	0.551 (0.542-0.56)	<0.001	<0.001	<0.001	<0.001	<0.001	<0.001
Stooping	0.532 (0.525-0.538)	0.517 (0.513-0.522)	0.518 (0.513-0.523)	0.560 (0.551-0.569)	0.561 (0.552-0.570)	0.566 (0.556-0.576)	0.554 (0.543-0.564)	<0.001	<0.001	<0.001	0.029	0.031	0.002
Lifting 5kg	0.583 (0.568-0.597)	0.552 (0.541-0.564)	0.562 (0.550-0.575)	0.617 (0.600-0.634)	0.627 (0.610-0.644)	0.637 (0.619-0.654)	0.627 (0.610-0.645)	<0.001	<0.001	<0.001	<0.001	0.023	0.187

Picking	0.563 (0.539-0.588)	0.547 (0.527-0.567)	0.548 (0.527-0.569)	0.614 (0.584-0.644)	0.614 (0.584-0.645)	0.627 (0.596-0.657)	0.612 (0.581-0.643)	<0.001	<0.001	<0.001	0.141	0.123	0.248
Arm	0.550 (0.538-0.563)	0.531 (0.521-0.540)	0.538 (0.527-0.549)	0.570 (0.554-0.586)	0.577 (0.561-0.594)	0.587 (0.570-0.603)	0.572 (0.555-0.589)	<0.001	<0.001	<0.001	0.001	0.028	0.033
Summary	0.537 (0.533-0.541)	0.516 (0.514-0.519)	0.522 (0.518-0.525)	0.565 (0.559-0.572)	0.571 (0.564-0.578)	0.583 (0.576-0.591)	0.575 (0.566-0.583)	<0.001	<0.001	<0.001	<0.001	<0.001	0.020

Abbreviations: AUC (95%CI), area under the curve (95% confidence interval); GLIS, the Global Leadership Initiative in Sarcopenia; H/A/M, low handgrip strength, low appendicular skeletal muscle mass index and low muscle-specific strength; AWGS 2019, the Asian Working Group for Sarcopenia 2019 framework; ADL, Activities of Daily Living; IADL, Instrumental Activities of Daily Living; Other, other functional capacity items; P1, AWGS 2019 vs. GLIS H/M; P2, GLIS HAM vs. GLIS H/M; P3, GLIS HA vs. GLIS H/M; P4 GLIS M vs. GLIS H/M; P5 GLIS HA/M vs. GLIS H/M; P5, GLIS A/M vs. GLIS H/M.

Table S4. Associations of different sarcopenia criteria with functional capacity in the outcome study population

	Adjusted OR (95%CI)[1]							
	no/events	AWGS 2019	GLIS HAM	GLIS HA	GLIS M	GLIS HA/M	GLIS H/M	GLIS A/M
ADL	11241/1416	2.142 (1.803-2.538)	2.297 (1.781-2.947)	1.932 (1.551-2.397)	2.024 (1.762-2.324)	2.059 (1.791-2.365)	2.164 (1.901-2.464)	1.831 (1.584-2.116)
Dressing	11241/320	3.320 (2.479-4.414)	3.369 (2.250-4.961)	2.785 (1.926-3.977)	2.972 (2.290-3.849)	3.111 (2.389-4.045)	3.218 (2.501-4.142)	2.879 (2.168-3.819)
Bathing	11241/356	2.996 (2.266-3.935)	2.993 (2.031-4.341)	2.579 (1.815-3.622)	2.838 (2.215-3.630)	3.004 (2.337-3.857)	3.274 (2.576-4.165)	2.784 (2.127-3.642)
Eating	11241/130	5.442 (3.620-8.183)	4.585 (2.714-7.744)	3.944 (2.409-6.459)	3.938 (2.652-5.848)	4.441 (2.955-6.675)	5.976 (3.955-9.031)	2.922 (1.850-4.614)
Bed	11241/309	1.983 (1.457-2.700)	2.569 (1.717-3.844)	2.056 (1.418-2.980)	2.071 (1.584-2.708)	2.061 (1.570-2.706)	2.181 (1.685-2.823)	1.911 (1.425-2.564)
Toilet	11241/938	2.094 (1.718-2.551)	2.052 (1.534-2.743)	1.839 (1.427-2.371)	2.041 (1.732-2.405)	2.102 (1.783-2.478)	2.221 (1.903-2.592)	1.841 (1.550-2.185)
Urination	11241/331	1.672 (1.219-2.294)	2.131 (1.399-3.244)	1.683 (1.144-2.476)	2.057 (1.589-2.663)	2.009 (1.546-2.610)	2.198 (1.715-2.816)	1.673 (1.267-2.210)
IADL	11241/1943	1.820 (1.551-2.136)	2.001 (1.577-2.538)	1.726 (1.411-2.110)	1.986 (1.755-2.249)	2.017 (1.781-2.283)	1.964 (1.750-2.206)	1.744 (1.532-1.985)
Money	11241/1129	1.882 (1.559-2.271)	1.975 (1.507-2.589)	1.718 (1.358-2.174)	1.892 (1.624-2.205)	1.923 (1.650-2.242)	1.916 (1.660-2.212)	1.655 (1.406-1.948)
Medication	11241/549	1.853 (1.440-2.385)	1.855 (1.299-2.650)	1.673 (1.222-2.290)	2.079 (1.690-2.558)	2.133 (1.731-2.629)	1.958 (1.607-2.385)	1.987 (1.589-2.484)
Shopping	11241/605	2.259 (1.802-2.832)	2.248 (1.645-3.072)	1.854 (1.401-2.453)	2.223 (1.827-2.705)	2.241 (1.837-2.735)	2.228 (1.845-2.691)	1.880 (1.510-2.341)
Meal	11241/532	2.227 (1.754-2.827)	2.132 (1.535-2.963)	1.662 (1.231-2.243)	2.362 (1.926-2.896)	2.312 (1.878-2.845)	2.701 (2.216-3.293)	1.835 (1.461-2.304)
Housework	11241/588	2.390 (1.905-2.997)	2.794 (2.056-3.799)	2.142 (1.619-2.834)	2.602 (2.141-3.164)	2.589 (2.123-3.158)	2.806 (2.322-3.391)	2.050 (1.652-2.544)
Other	11241/7182	2.326 (1.909-2.833)	2.603 (1.839-3.685)	1.946 (1.516-2.497)	2.060 (1.816-2.336)	2.056 (1.819-2.324)	2.118 (1.895-2.367)	1.759 (1.572-1.968)
Jogging 1km	11241/5386	2.079 (1.768-2.444)	1.973 (1.515-2.571)	1.777 (1.445-2.186)	1.971 (1.764-2.203)	2.010 (1.802-2.242)	2.092 (1.894-2.311)	1.798 (1.619-1.997)
Walking 1km	11241/923	2.391 (1.970-2.901)	2.528 (1.912-3.342)	2.129 (1.664-2.724)	2.178 (1.848-2.567)	2.234 (1.893-2.636)	2.384 (2.040-2.785)	1.964 (1.651-2.337)
Walking 100m	11241/157	3.060 (2.063-4.539)	3.349 (1.977-5.673)	2.344 (1.417-3.877)	3.586 (2.497-5.148)	3.415 (2.363-4.936)	3.492 (2.443-4.993)	3.428 (2.317-5.072)
Chair	11241/2617	1.963 (1.686-2.286)	1.881 (1.486-2.381)	1.686 (1.384-2.054)	1.841 (1.638-2.069)	1.874 (1.669-2.104)	1.919 (1.725-2.136)	1.634 (1.454-1.836)
Climbing	11241/4069	1.935 (1.668-2.244)	2.190 (1.729-2.774)	1.868 (1.544-2.260)	1.819 (1.633-2.028)	1.854 (1.666-2.064)	1.883 (1.707-2.078)	1.542 (1.387-1.715)
Stooping	11241/2866	1.737 (1.494-2.019)	2.129 (1.690-2.683)	1.650 (1.360-2.003)	1.909 (1.705-2.138)	1.873 (1.673-2.097)	1.766 (1.591-1.960)	1.676 (1.496-1.877)
Lifting 5kg	11241/838	2.596 (2.131-3.162)	3.262 (2.478-4.294)	2.706 (2.123-3.448)	2.270 (1.911-2.695)	2.393 (2.012-2.847)	2.401 (2.038-2.829)	2.087 (1.729-2.519)
Picking	11241/261	2.037 (1.453-2.856)	2.782 (1.801-4.297)	2.041 (1.358-3.067)	2.365 (1.776-3.151)	2.276 (1.700-3.048)	2.367 (1.793-3.125)	2.037 (1.486-2.793)
Arm	11241/868	2.154 (1.752-2.649)	2.565 (1.919-3.429)	2.273 (1.762-2.932)	1.828 (1.540-2.169)	1.917 (1.615-2.275)	1.953 (1.664-2.292)	1.589 (1.326-1.904)
Summary	11241/7431	2.417 (1.965-2.973)	2.670 (1.849-3.855)	2.027 (1.559-2.635)	2.102 (1.847-2.393)	2.109 (1.859-2.393)	2.157 (1.924-2.418)	1.809 (1.613-2.028)

Abbreviations: OR (95%CI), odds ratio (95% confidence interval); AWGS 2019, the Asian Working Group for Sarcopenia 2019 framework; GLIS, the Global Leadership Initiative in Sarcopenia; H/A/M, low handgrip strength, low appendicular skeletal muscle mass index and low muscle-specific strength; ADL, activities of daily living; IADL, instrumental activities of daily living; Other, other functional capacity items.

[1] Adjusted for age (continuous), sex (reference = women) and body mass index (continuous). Odds ratios are calculated using the Firth's bias-reduced Logistic regression models.

Table S5. Baseline characteristics of the external validation population

		GLIS H/M-defined sarcopenia		
Characteristics	Overall (n=504)	No (n=291)	Yes (n=213)	*P*
Age, years	48.2±13.2[1]	45.0±11.9	52.5±13.6	<0.001
Sex, men	247 (49.0)[2]	139 (47.8)	108 (50.7)	0.574
Body height, m	1.6±0.1	1.6±0.1	1.6±0.1	<0.001
Body weight, kg	63.3±15.5	65.2±16.5	60.8±13.8	0.002
Body mass index, kg/m^2	24.1±5.5	24.5±5.8	23.7±5.0	0.092
Body mass index group				0.270
Underweight	31 (6.2)	13 (4.5)	18 (8.5)	
Normal	272 (54.0)	156 (53.6)	116 (54.5)	
Overweight	123 (24.4)	75 (25.8)	48 (22.5)	
Obese	78 (15.5)	47 (16.2)	31 (14.6)	
Smoking				0.401
None smoker	325 (64.5)	193 (66.3)	132 (62.0)	
Current smoker	75 (14.9)	44 (15.1)	31 (14.6)	
Former smoker	104 (20.6)	54 (18.6)	50 (23.5)	
Drinking, yes	71 (14.1)	36 (12.4)	35 (16.4)	0.244
Diabetes, yes	74 (14.7)	30 (10.3)	44 (20.7)	0.002
Hypertension, yes	395 (78.4)	234 (80.4)	161 (75.6)	0.234
Anemia, yes	323 (64.1)	195 (67.0)	128 (60.1)	0.132
CKD stage, V vs. IV	377 (74.8)	220 (75.6)	157 (73.7)	0.704
Handgrip strength, kg	26.7±11.0	30.9±11.2	20.9±7.7	<0.001
Handgrip strength, low	173 (34.3)	0 (0.0)	173 (81.2)	<0.001
ASMI, kg/m^2	7.1±1.5	7.2±1.5	7.0±1.4	0.051
ASMI, low	60 (11.9)	22 (7.6)	38 (17.8)	0.001
ASM, kg	18.8±4.9	19.4±5.1	18.1±4.6	0.003
Chair stand test, s	11.0±3.9	9.7±2.1	12.7±4.9	<0.001
Chair stand test, impaired	146 (29.0)	39 (13.4)	107 (50.2)	<0.001
Muscle specific strength	0.9±0.4	1.0±0.4	0.8±0.3	<0.001
Muscle specific strength, low	104 (20.6)	0 (0.0)	104 (48.8)	<0.001
AWGS 2019, grade				<0.001
Not sarcopenia	411 (81.5)	291 (100.0)	120 (56.3)	
Sarcopenia	83 (16.5)	0 (0.0)	83 (39.0)	
Severe sarcopenia	10 (2.0)	0 (0.0)	10 (4.7)	
AWGS 2019, yes	93 (18.5)	0 (0.0)	93 (43.7)	<0.001
GLIS HAM, yes	13 (2.6)	0 (0.0)	13 (6.1)	<0.001
GLIS HA, yes	29 (5.8)	0 (0.0)	29 (13.6)	<0.001
GLIS M, yes	104 (20.6)	0 (0.0)	104 (48.8)	<0.001
GLIS HA/M, yes	120 (23.8)	0 (0.0)	120 (56.3)	<0.001
GLIS H/M, yes	213 (42.3)	0 (0.0)	213 (100.0)	<0.001
GLIS A/M, yes	142 (28.2)	22 (7.6)	120 (56.3)	<0.001
Functional capacity				

ADL all, continuous	0.3±0.9	0.2±0.8	0.4±1.0	0.099
ADL all, ≥1	73 (14.5)	36 (12.4)	37 (17.4)	0.148
Dressing	19 (3.8)	7 (2.4)	12 (5.6)	0.100
Bathing	42 (8.3)	22 (7.6)	20 (9.4)	0.568
Eating	19 (3.8)	10 (3.4)	9 (4.2)	0.824
Bed	11 (2.2)	3 (1.0)	8 (3.8)	0.078
Toilet	22 (4.4)	9 (3.1)	13 (6.1)	0.158
Urination	31 (6.2)	15 (5.2)	16 (7.5)	0.368
IADL all, continuous	0.5±1.1	0.3±0.8	0.7±1.5	<0.001
IADL all, ≥1	108 (21.4)	46 (15.8)	62 (29.1)	<0.001
Money	42 (8.3)	24 (8.2)	18 (8.5)	1.000
Medication	14 (2.8)	7 (2.4)	7 (3.3)	0.749
Shopping	28 (5.6)	7 (2.4)	21 (9.9)	0.001
Meal	43 (8.5)	11 (3.8)	32 (15.0)	<0.001
Housework	81 (16.1)	29 (10.0)	52 (24.4)	<0.001
Other FC items, continuous	2.3±2.6	1.6±1.8	3.3±3.2	<0.001
Other FC items, ≥1	382 (75.8)	197 (67.7)	185 (86.9)	<0.001
Jogging 1km	315 (62.5)	157 (54.0)	158 (74.2)	<0.001
Walking 1km	92 (18.3)	31 (10.7)	61 (28.6)	<0.001
Walking 100m	14 (2.8)	1 (0.3)	13 (6.1)	<0.001
Chair	44 (8.7)	12 (4.1)	32 (15.0)	<0.001
Climbing	282 (56.0)	128 (44.0)	154 (72.3)	<0.001
Stooping	99 (19.6)	39 (13.4)	60 (28.2)	<0.001
Lifting 5kg	144 (28.6)	57 (19.6)	87 (40.8)	<0.001
Picking	18 (3.6)	6 (2.1)	12 (5.6)	0.059
Arm	21 (4.2)	1 (0.3)	20 (9.4)	<0.001
Summary all, continuous	3.1±4.0	2.2±2.6	4.3±5.0	<0.001
Summary all, ≥1	395 (78.4)	208 (71.5)	187 (87.8)	<0.001

Abbreviations: GLIS, the Global Leadership Initiative in Sarcopenia; CKD, chronic kidney disease; ASMI, appendicular skeletal muscle mass index; ASM, appendicular skeletal muscle mass; AWGS 2019, the Asian Working Group for Sarcopenia 2019 framework; H/A/M, low handgrip strength, low appendicular skeletal muscle mass index and low muscle-specific strength; ADL, Activities of Daily Living; IADL, Instrumental Activities of Daily Living (IADL).

[1] Mean ± standard deviation, all such values.

[2] Number (percentage), all such values.

Table S6. Performance of different sarcopenia criteria for diagnosing functional capacity outcomes in the external validation population

	AUC (95%CI)							Delong's test (Reference = GLIS H/M)					
	AWGS 2019	GLIS HAM	GLIS HA	GLIS M	GLIS HA/M	GLIS H/M	GLIS A/M	*P1*	*P2*	*P3*	*P4*	*P5*	*P6*
ADL all	0.536 (0.483-0.589)	0.517 (0.490-0.544)	0.522 (0.487-0.558)	0.532 (0.477-0.586)	0.537 (0.480-0.594)	0.549 (0.487-0.611)	0.511 (0.454-0.569)	0.641	0.307	0.390	0.515	0.632	0.151
IADL all	0.583 (0.536-0.630)	0.531 (0.505-0.556)	0.528 (0.498-0.559)	0.587 (0.538-0.635)	0.584 (0.534-0.634)	0.596 (0.544-0.649)	0.568 (0.517-0.619)	0.570	0.015	0.011	0.672	0.574	0.239
Other FC	0.584 (0.554-0.613)	0.517 (0.508-0.526)	0.522 (0.503-0.540)	0.587 (0.556-0.619)	0.592 (0.557-0.627)	0.627 (0.582-0.672)	0.589 (0.549-0.628)	0.034	<0.001	<0.001	0.043	0.057	0.083
Summary	0.577 (0.546-0.608)	0.516 (0.508-0.525)	0.519 (0.499-0.539)	0.585 (0.552-0.617)	0.588 (0.552-0.623)	0.617 (0.570-0.665)	0.580 (0.539-0.621)	0.056	<0.001	<0.001	0.116	0.122	0.118

Abbreviations: AUC (95%CI), area under the curve (95% confidence interval); GLIS, the Global Leadership Initiative in Sarcopenia; H/A/M, low handgrip strength, low appendicular skeletal muscle mass index and low muscle-specific strength; AWGS 2019, the Asian Working Group for Sarcopenia 2019 framework; ADL, Activities of Daily Living; IADL, Instrumental Activities of Daily Living; Other FC, other functional capacity items; P1, AWGS 2019 vs. GLIS H/M; P2, GLIS HAM vs. GLIS H/M; P3, GLIS HA vs. GLIS H/M; P4 GLIS M vs. GLIS H/M; P5 GLIS HA/M vs. GLIS H/M; P5, GLIS A/M vs. GLIS H/M.

Table S7. Associations of different sarcopenia criteria with functional capacity in the external validation population

		Adjusted OR (95%CI)[1]						
	no/events	AWGS 2019	GLIS HAM	GLIS HA	GLIS M	GLIS HA/M	GLIS H/M	GLIS A/M
ADL	504/73	1.951 (1.056-3.603)	3.724 (1.119-12.390)	2.665 (1.062-6.686)	1.812 (0.988-3.325)	1.930 (1.065-3.497)	1.770 (1.056-2.966)	1.415 (0.781-2.562)
IADL	504/108	2.993 (1.773-5.053)	6.268 (2.024-19.413)	2.626 (1.164-5.925)	3.059 (1.826-5.124)	2.873 (1.724-4.787)	2.476 (1.571-3.901)	2.265 (1.368-3.750)
Other FC	504/382	3.796 (1.721-8.372)	8.051 (0.46-140.972)	2.276 (0.699-7.415)	3.645 (1.772-7.499)	3.327 (1.727-6.411)	2.889 (1.781-4.685)	2.663 (1.486-4.774)
Summary	504/395	3.371 (1.524-7.455)	6.917 (0.399-119.826)	2.047 (0.628-6.671)	3.720 (1.749-7.911)	3.313 (1.679-6.536)	2.744 (1.665-4.522)	2.523 (1.384-4.599)

Abbreviations: OR (95%CI), odds ratio (95% confidence interval); AWGS 2019, the Asian Working Group for Sarcopenia 2019 framework; GLIS, the Global Leadership Initiative in Sarcopenia; H/A/M, low handgrip strength, low appendicular skeletal muscle mass index and low muscle-specific strength; ADL, activities of daily living; IADL, instrumental activities of daily living; Other FC, other functional capacity items.

[1] Adjusted for age (continuous), sex (reference = women) and body mass index (continuous). Odds ratios are calculated using the Firth's bias-reduced Logistic regression models.

Figure S1

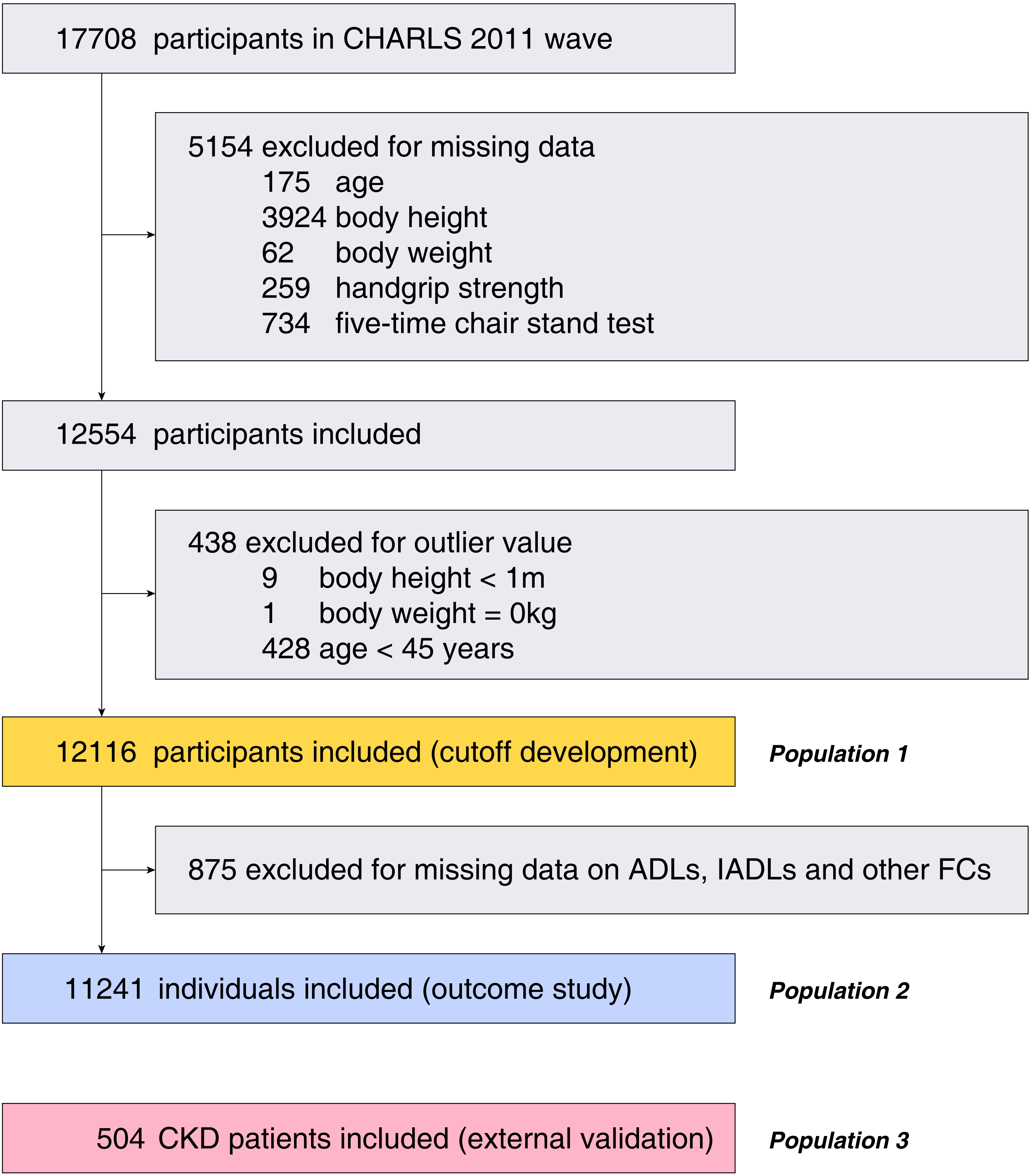
17708 participants in CHARLS 2011 wave
5154 excluded for missing data
175 age
3924 body height
62 body weight
259 handgrip strength
734 five-time chair stand test
12554 participants included
438 excluded for outlier value
9 body height < 1m
1 body weight = 0kg
428 age < 45 years
12116 participants included (cutoff development)
Population 1
875 excluded for missing data on ADLs, IADLs and other FCs
11241 individuals included (outcome study)
Population 2
504 CKD patients included (external validation)
Population 3
